# Ocular Dominance and Visual Function Testing

**DOI:** 10.1155/2013/238943

**Published:** 2013-11-11

**Authors:** D. Lopes-Ferreira, H. Neves, A. Queiros, M. Faria-Ribeiro, S. C. Peixoto-de-Matos, J. M. González-Méijome

**Affiliations:** Clinical & Experimental Optometry Research Laboratory, Center of Physics, University of Minho, Gualtar, 4710-057 Braga, Portugal

## Abstract

*Purpose*. To show the distribution of ocular dominance as measured with sensory and eye sighting methods and its potential relationship with high and low contrast LogMAR visual acuity in presbyopic subjects. *Method*. Forty-four presbyopes (48.5 ± 3.5 years) participated in this study. Ocular dominance was determined by eye sighting (hole-in-card) and sensorial (+1.50 D lens induced blur) methods. According to the dominance detected with each method (RE: right eye or LE: left eye), patients were classified in dominance type 1 (RE/RE), type 2 (RE/LE), type 3 (LE/RE) and type 4 (LE/LE). *Results*. Baseline refractive error (MSE) was RE:−0.36 ± 1.67 D and LE:−0.35 ± 1.85 D (*P* = 0.930). RE was the dominant eye in 61.4% and 70.5% of times as obtained from sensorial and sighting methods, respectively. Most frequent dominance was of type 1 (52.3%), in this case the RE showed statistically significant better distance low contrast LogMAR VA (0.04 LogMAR units) compared to the LE (*P* < 0.05). *Conclusions*. The dominance was more frequent in RE in this sample. The eye sighting and sensorial methods to define ocular dominance agreed in more than half of cases. Amount of MSE was not significantly different between dominant and non-dominant eye. But in case of right dominance, the RE presented better distance low contrast VA compared to the LE.

## 1. Introduction

The concept of ocular dominance consists in a tendency to prefer visual input from one eye with which subjects are more accurate and images appear clearer, more stabilized, and perhaps larger [1–4]. Recent data also suggests that the dominant eye has perceptual processing priority [5].

The prevailing view in the literature suggests that there is a single dominant eye for each person [2]. For example, the motivation for two recent fMRI studies was, in part, to find the neural basis of a “unitary dominant eye” [2, 6]. However, it is also known that the dominant eye is in part related to cerebral laterality (significantly higher cortical activation in response to the dominant eye than the non-dominant) [7] and to hand laterality [8].

 Determination of dominant eye is dependent on the test used [2, 9, 10] and gaze angle [11]. The most common method reported in the literature is the “hole-in-card” test that determines the ocular preference (sighting eye dominance) by using the hands. This raises the question of hand dominance interfering with the result [12]. Despite this, it has been described that sighting eye dominance is related with the dominant eye as determined by binocular rivalry (called “sensory” eye) though the controversy over equivalence of sighting and sensory eye dominance remains unresolved [13]. 

In monovision, the dominant eye is usually corrected for distance and the non-dominant eye for near, based on the hypothesis that the non-dominant eye will be more easily suppressed by the relatively blurred image in the fellow eye for distance [14, 15]. Eye dominance is recognized as one of the important factors in monovision success [16]. Handa et al. have shown that binocular summation only occurs in monovision when people with strong ocular dominance are fitted with the near vision lens in the non-dominant eye [17].

However, according to Robboy et al. sighting dominance alone is not an adequate measure of ocular dominance [18]. The method of resistance to blur is another method for determination of ocular dominance based on the assumption that it is easier to suppress blur in the non-dominant eye than in the dominant eye [16].

The aim of this study was to assess ocular dominance through an eye sighting method “hole-in-card” and sensory method (resistance to +1.5 D blur) and to evaluate their agreement and the potential relationship with visual acuity in high and low contrast for distant and near vision in presbyopic candidates to wear multifocal soft contact lenses.

## 2. Methods

For this study, 44 healthy presbyopic patients were recruited, 29 females and 15 males with spherical equivalent refractive error (MSE ± SD) of −0.36 D ± 1.67 and −0.33 D ± 1.80 in right and left eye (*P* = 0.93), respectively, and refractive astigmatism ≤1.00 D. Patient's age was between 41 and 56 years (mean 48.5 ± 3.5). The experiments were conducted at the Clinical and Experimental Optometry Research Lab (CEORLab, Minho University, Braga, Portugal). The purpose and all the procedures were explained to every patient, and an informed consent was obtained according to the declaration of Helsinki. The study was approved by Scientific Committee of the School of Sciences at the University of Minho (Portugal).

Patients underwent a complete optometric examination including evaluation of high and low contrast LogMAR distance and near visual acuity and retinoscopy followed by subjective refraction with an end-point of maximum plus for better distance visual acuity. None of the patients was amblyopic or had anisometropia higher than 0.50 D, had not undergone any ocular surgery, and were not under ocular or systemic medications susceptible to affect the visual system. Sighting dominance was determined by instructing the patient to fixate one letter (corresponding to 7/10) at distance through a “hole” between his/her hands having their arms outstretched to warrant that the hole is in the middle line of the patient's body. Then, the subject's eyes were alternately occluded briefly and the subject was asked to report when the target was visible. Dominant eye was the eye that could maintain the fixed letter centered in the hole or close, being the contralateral eye occluded.

In turn, sensory dominance was determined with patient fixating a high contrast LogMAR visual acuity chart at 4 meters to the line immediately larger than the best patient's visual acuity while a +1.50 D lens was alternated in front of each patient's eye during few seconds. The dominant eye was the one in which the subject reported more blurred vision with the positive lens under binocular conditions. 

Best corrected distance VA (BCDVA) was measured as recommended at 4 meters with the Logarithmic Visual Acuity Chart “ETDRS” (Precision Vision, IL). The ETDRS chart of distance is constituted by 14 lines with 5 letters each and can measure VA between 1.0 LogMAR units (that is equivalent to 0.1 in decimal scale) and −0.3 LogMAR units (2.0 in decimal scale). The line of 20/20 (or 1.0 in decimal scale) is equivalent to 0.0 (zero) in LogMAR scale. Each letter read means −0.02, and so VA is better if it is more negative or less positive. VA was evaluated under high (100%) (CAT No 2110) and low (10%) contrast (CAT No 2153) conditions using Cabinet Illuminator No 2425.

Best corrected near VA (BCNVA) was measured at a 40 cm distance with Logarithmic Visual Acuity Chart 2000 “New ETDRS” (Chart “1”-CAT No 2106), as recommended, for high (100%) contrast and with Chart “2” (CAT No 2117) for low (10%) contrast conditions. Similarly to the distance chart, near chart is constituted by 17 lines with 5 letters each and can measure VA between 1.4 LogMAR units (that is equivalent to 0.05 in decimal scale) and 0.3 (2.0 in decimal scale). VA at near and distance were scored following the same procedure. VA measures were taken monocularly and binocularly in all referred conditions. Room luminance was kept at photopic levels (85 cd/m^2^) during the whole examination. According to the dominant eye identified (RE: right eye or LE: left eye) with each method (sighting/sensorial), patients were classified in dominance type 1 (RE/RE) or dominance type 4 (LE/LE) if both methods agree that right or left eye was dominant, respectively, and dominance type 2 (RE/LE) or dominance type 3 (LE/RE) if sighting method demonstrated that left eye was dominant and sighting method demonstrated that right eye was dominant or vice versa, respectively.

Statistical analysis was conducted using IBM SPSS Statistics Software, for Windows, Version 19 (SPSS Inc, Chicago, IL, USA). Normal distribution of data within each dominance type was evaluated using Shapiro-Wilk test. One-way ANOVA with Bonferroni posthoc correction was used for comparisons between dominance types with normal distributions and with independent samples Mann-Whitney test for distributions that did not follow a normal distribution. To compare variables within each dominance type, paired sample *t*-test and Wilcoxon signed ranks test were used to compare normally and nonnormally distributed data, respectively. The kappa statistic (*κ* statistic) was used to assess the agreement between the two tests of dominance evaluation. A *P* value ≤ 0.05 was considered to be significant.

## 3. Results

The right eye was the dominant eye in 61.4% and 70.5% of patients by sensory and sighting method, respectively. Both methods of ocular dominance determination agreed in 72.7% of cases. Right dominance was more frequent than left as the proportion of individuals with dominance type 1 (52.3%) was higher than with dominance type 4 (20.5%).

Table 3 presents the mean values of monocular and binocular LogMAR visual acuity (VA) for each dominance type, for distance and near vision under high and low contrast conditions.

Table 1 shows differences in MSE and VA values between the right and left eyes within each dominance type. In this case, differences between VA of both eyes were not statistically significant, except in the case of right dominance determined by both methods (dominance type 1) in which distance VA measured under low contrast condition manifested statistically significant differences between dominant and non-dominant eye. In this case, the difference was of −0.04 LogMAR units (*P* = 0.045), with the right eye (dominant) having a better VA than the left eye (non-dominant). 

**Figure 1:**
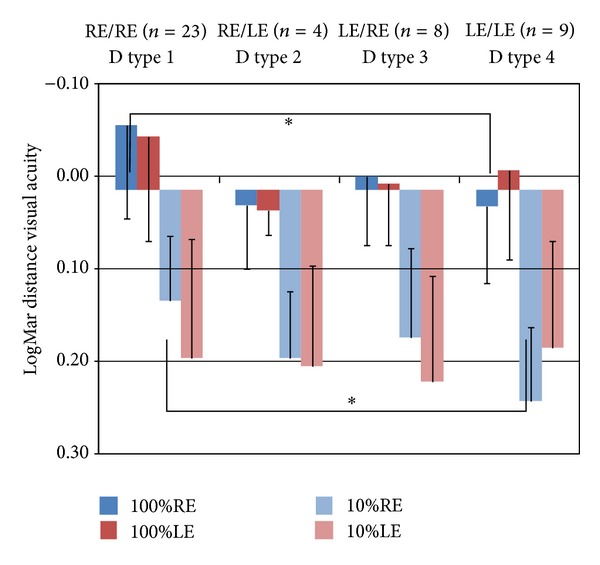
Mean best corrected visual acuity in distance vision and standard deviation for every dominance type (1 to 4): high contrast (100%), low contrast (10%) for right (RE) and left (LE). The values of VA are represented in LogMAR units. *P < 0.05 in comparison.

As we can see in Figure 1 where there is a comparison between dominance types 1 and 4, we found slightly better distance VA in the dominant eye for high and low contrast conditions. 

Numerical differences are referred to and *P* values of comparisons are presented in Table 4 showing differences of −0.08 ± 0.03 (*P* = 0.032) and −0.10 ± 0.03 (*P* = 0.007), respectively, for high and low contrast conditions and negative sign means that when dominant eye is the right (dominance type 1) it has better LogMAR VA. This difference represents 4 additional letters and a whole line of distance LogMAR VA in relation to non-dominant eye (the left eye-dominance type 4). At near we also found differences of −0.08 ± 0.07 LogMAR units between the low contrast VA in right eye between dominance types 1 and 4 (*P* = 0.049).

The agreement between the two tested methods to determine ocular dominance was evaluated through the kappa statistic. It revealed a weak statistical correlation between methods to determine ocular dominance in this population of presbyopes (*κ* statistic = 0.399).

## 4. Discussion

 The present study shows the distribution of ocular dominance in presbyopic patients, and it was showed that right eye was more frequently the dominant eye (52.3%). Unanimous results published in the literature report that the right eye is the dominant eye in the majority of patients [1, 12, 18, 22, 23, 26–28].  Table 2 presents an overview on the ocular dominance experiments conducted by other authors. It is evident that our relative frequencies of ocular dominance for the right eye are very close to the mean value obtained as the arithmetic mean of eleven previous studies (50.7%). The methods chosen for sighting and sensorial determination of ocular dominance were described as being very useful, with fewer uncertain results, and showed 40% of agreement between them, for patients with mean age 43.7 ± 5.9 years in a previous study [24]. Our results showed a higher level of agreement between methods (52.3%). Despite this we could not find a significant statistical correlation between the motor and sensory ocular dominance tests used (*κ* statistic < 0.5) as in the study of Seijas et al. [24].

We further explored the relationship between ocular dominance and VA, and we could verify that when right eye was the dominant eye, its visual acuity was slightly better than the left non-dominant eye (Table 1). It is somewhat intuitive to think that the preferred eye is coincidental with the better eye in terms of visual acuity, but in a previous study [12], it was shown that the relation between ocular dominance and ocular preference did not exist.

Our results might be particularly relevant in visual compensation under low contrast conditions. Despite low contrast, VA measurement is not common in clinical practice, as exposed above; it can be useful in helping to do a most correct determination of ocular dominance in order to arrive to better visual performance results. Ocular dominance was considered as being independent of refractive error [23, 29], and our present data are in agreement with this previous findings as we did not find differences in spherical equivalent refraction (MSE) between dominant and non-dominant eye in each dominance type. This fact contrasts with recent findings reporting that the dominant eye had a greater myopic refractive error and longer axial length than the non-dominant eye, particularly in subjects with higher amounts than 1.75 D of anisometropia in which the dominant eye was always more myopic than the non-dominant eye [20]. However, none of our patients was anisometropic.

A limitation of the present study is the reduced number of patients in three of the 4 dominance types established according to their dominance results with both measuring techniques. However, this is the result of the stratification of the initially enrolled subjects and represents the normal presbyopic population. However, it could limit our ability to find significant differences or correlations between some of the parameters evaluated and, in the future, larger samples must be enrolled to warrant a minimum of patients in each dominance type to reevaluate some of the relationships. 

The present results might be useful for its application in the field of intraocular lens implantation for monovision in presbyopic patients [21, 30]. Further, our results point in the direction of other authors [24] who reported that it would be necessary to carry out more than one test of dominance, privileging the joint evaluation of sensory methods as a mean of evaluating the magnitude of ocular dominance [21, 24]. Considering the existent difference between binocular summation in a weak ocular dominance or in a strong ocular dominance [17], the use of a combination of methods in the determination of the ocular dominance results in a more consistent information when it comes to taking some decisions such as the type of IOL to be implanted in dominant or non-dominant eye or the profile of corneal ablation.

In summary, this study showed a relationship between the dominant eye and its higher visual capabilities in terms of resolution of low contrast details. This might be of relevance in clinical terms and enhances the critical relevance of determining dominance correctly when deciding which contact lens or surgical procedures to apply to each eye. This study shows that a correct election of dominance might be critical for optimization of near and distance visual tasks under low contrast conditions. According to the present results, visual acuity plays an important role in choosing the dominant eye. These differences have a greater expression in the especially more demanding near and distance visual tasks (under low contrast conditions).

## Figures and Tables

**Table 1:** Differences and standard deviation of spherical equivalent refraction (MSE) and monocular (right minus left) best corrected visual acuity (BCVA) measurements (LogMAR units) for distance (BCDVA) and for near conditions (BCNVA) under high (100%) and low (10%) contrast within each dominance type group. Bold represents statistically significant differences. Negative values represent better VA for the right eye.

Dominance type	Right eye versus left eye				
	MSE (mean ± SD) *P* value	100% BCDVA (mean ± SD) *P* value	10% BCDVA (mean ± SD) *P* value	100% BCNVA (mean ± SD) *P* value	10% BCNVA (mean ± SD) *P* value
1 (*n* = 23)	−0.11 ± 0.38 0.150^§^	−0.01 ± 0.09 0.471^§^	−0.04 ± 0.10 **0.045** ^¥^	−0.01 ± 0.10 0.556^§^	−0.02 ± 0.09 0.304^¥^
2 (*n* = 4)	−0.28 ± 0.26 0.102^§^	−0.01 ± 0.04 0.655^§^	0.00 ± 0.06 1.000^§^	0.01 ± 0.04 0.581^§^	−0.03 ± 0.05 0.180^§^
3 (*n* = 8)	0.31 ± 0.67 0.173^§^	−0.01 ± 0.06 0.666^§^	−0.03 ± 0.09 0.705^§^	0.10 ± 0.11 0.034^§^	0.06 ± 0.10 0.144^§^
4 (*n* = 9)	−0.01 ± 0.96 0.237^§^	0.03 ± 0.07 0.101^§^	0.02 ± 0.12 0.622^§^	−0.13 ± 0.46 0.599^§^	0.06 ± 0.10 0.107^§^

^*¥*^Paired sample *t*-test; ^§^Wilcoxon signed ranks test.

**Table 2:** Summary of relative frequencies of ocular dominance obtained by different authors with different methods.

Author (year)	Method used	*n*	Mean/range of age	Right dominance (%)	Left dominance (%)	Uncertain (%)
Porac and Coren (1976) [1]	Hole-in-card test	—	—	65	33	2

Newman et al. (1985) [19]	Hole-in-card test	298	7–11	65.6	34.4	—

Rombouts et al. (1996) [6]	fMRI	26	23.3 ± 3.5	53.8	30.8	15.4

Pointer (2001) [12]	Hole-in-card test	200	13.1 ± 2.4	69.5	30.5	—
		200	45.1 ± 13.8	76	24	—

Cheng et al. (2004) [20]	Hole-in-card test	50	30.3 ± 9.5	63.6	36.4	—
	Convergence near-point test			43.6	32.7	23.6

Handa et al. (2004) [21]	Hole-in-card test	20	60	75	25	—

Ehrenstein et al. (2005) [22]	Hole-in-card test	103	24.1 ± 4.5	68	32	—

Shneor and Hochstein (2006) [5]	Hole-in-card test	21	19–57	61.9	39.1	—

Chia et al. (2007) [23]	Hole-in-card test	543	12–13	58	30	12

Seijas et al. (2007) [24]	Hole-in-card test	26	26 ± 2	50	50	—
		25	43.7 ± 5.9	60	40	—
	Finger pointing			46.2	30.8	23
				40	48	12
	Kaleidoscope			69.2	30.8	—
				60	40	—
	Convergence near-point test			33.3	37.5	29.2
				36	24	40
	Distance stereo			7.7	15.4	76.9
				4	4	92
	Haidinger test			38.5	23	38.5
				24	16	60

Rice et al. (2008) [25]	Hole-in-card test	46	42.5/(18–78)	60.9	39.1	—
	Convergence near-point test			37.0	41.3	21.7

Mean values				**50.7**	**31.5**	**17.8**

**Table 3:** Average monocular best corrected visual acuity values and standard deviation for every experimental viewing condition: distance, near, high contrast (100%) and low contrast (10%) for right (RE), left (LE), and both eyes (BE) and for each dominance type group (1 to 4). For *M* value, the maximum and minimum for each eye and dominance type group were also represented. The values of VA are represented in LogMAR units, MSE in diopters, and age in years.

	Dominance type			
	1 (*n* = 23)	2 (*n* = 4)	3 (*n* = 8)	4 (*n* = 9)
Age (mean ± SD)	49.0 ± 4.2	47.8 ± 1.9	46.5 ± 3.4	48.2 ± 2.8

MSE (mean ± SD) (min; max)				
RE	−0.11 ± 1.06 (−3.13; 0.75)	−1.19 ± 2.55 (−5.00; 0.38)	−0.12 ± 2.54 (−6.25; 1.50)	−0.85 ± 1.72 (−5.00; 1.25)
LE	0.00 ± 1.11 (−3.25; 2.13)	−0.91 ± 2.76 (−5.00; 0.88)	−0.44 ± 2.97 (−7.50; 1.75)	−0.83 ± 1.66 (−3.88; 1.63)

Distance LogMAR VA (mean ± SD)				
100%				
RE	−0.06 ± 0.09	0.02 ± 0.06	−0.01 ± 0.06	0.02 ± 0.07
LE	−0.05 ± 0.10	0.02 ± 0.02	−0.01 ± 0.06	−0.02 ± 0.08
BE	−0.08 ± 0.09	−0.06 ± 0.05	−0.08 ± 0.06	−0.06 ± 0.06
10%				
RE	0.11 ± 0.06	0.16 ± 0.06	0.14 ± 0.08	0.20 ± 0.07
LE	0.15 ± 0.11	0.16 ± 0.10	0.17 ± 0.10	0.18 ± 0.10
BE	0.05 ± 0.06	0.11 ± 0.07	0.06 ± 0.06	0.14 ± 0.18

Near LogMAR VA (mean ± SD)				
100%				
RE	0.08 ± 0.17	0.07 ± 0.07	0.15 ± 0.19	0.08 ± 0.18
LE	0.09 ± 0.17	0.06 ± 0.10	0.06 ± 0.12	0.21 ± 0.47
BE	0.03 ± 0.15	−0.06 ± 0.09	0.11 ± 0.12	−0.01 ± 0.16
10%				
RE	0.24 ± 0.22	0.23 ± 0.10	0.28 ± 0.15	0.32 ± 0.12
LE	0.26 ± 0.17	0.26 ± 0.09	0.22 ± 0.14	0.26 ± 0.16
BE	0.18 ± 0.17	0.19 ± 0.08	0.19 ± 0.21	0.22 ± 0.11

**Table 4:** Difference in patient's age, mean spherical equivalent (MSE), and monocular and binocular (both eyes, BE) best corrected visual acuity (BCVA) at high (100%) and low contrast (10%) for distance vision (BCDVA) and near vision (BCNVA) between dominance types. Bold represents statistically significant differences. Italic column (1-4) highlights true comparison between right (type 1: RE/RE) and left (type 4: LE/LE) dominance type. The negative sign represents better VA for the first dominance type in the comparison.

	1-2	1-3	*1-4 *	2-3	2-4	3-4
MSE (D)						
RE	1.076 ± 0.912 0.452*	0.015 ± 0.691 0.154*	0.740 ± 0.662 0.131*	−1.061 ± 1.031 1.000^+^	−0.336 ± 1.011 1.000^+^	0.725 ± 0.818 1.000^+^
LE	0.906 ± 0.991 0.945*	0.439 ± 0.751 0.571*	0.836 ± 0.719 0.097*	−0.468 ± 1.120 1.000^+^	−0.071 ± 1.099 0.588*	0.397 ± 0.889 0.193*

BCDVA (LogMAR)						
100% (RE)	−0.08 ± 0.04 0.087*	−0.05 ± 0.03 0.141*	*−0.08 ± 0.03 * 0.032*	0.03 ± 0.05 0.495*	−0.00 ± 0.05 0.938*	−0.03 ± 0.04 0.358*
10% (RE)	−0.06 ± 0.04 0.091*	−0.04 ± 0.03 0.632^+^	*−0.10 ± 0.03 * 0.007*	0.02 ± 0.04 0.862*	−0.04 ± 0.04 0.391*	−0.06 ± 0.03 0.043*
100% (LE)	−0.07 ± 0.05 0.132*	−0.05 ± 0.04 0.248*	−0.03 ± 0.03 0.388*	0.03 ± 0.05 0.301*	0.04 ± 0.05 0.349*	0.01 ± 0.04 0.772*
10% (LE)	−0.01 ± 0.06 0.458*	−0.02 ± 0.04 0.346*	−0.03 ± 0.04 0.243*	−0.01 ± 0.07 0.547*	−0.02 ± 0.07 0.698*	−0.02 ± 0.06 0.808*
100% (BE)	−0.02 ± 0.04 0.389*	−0.01 ± 0.03 0.508*	−0.02 ± 0.03 0.184*	0.02 ± 0.05 0.863*	0.00 ± 0.05 0.693*	−0.02 ± 0.04 0.497*
10% (BE)	−0.05 ± 0.05 0.119*	−0.01 ± 0.04 0.536*	*−0.09 ± 0.04 0.158* ^ +^	0.05 ± 0.06 0.441*	−0.04 ± 0.06 1.000*	−0.08 ± 0.05 0.460*

BCNVA (LogMAR)						
100% (RE)	0.01 ± 0.09 0.706*	−0.08 ± 0.07 0.221*	0.00 ± 0.07 0.916*	−0.08 ± 0.11 0.306*	−0.01 ± 0.10 0.938*	0.08 ± 0.09 0.358*
10% (RE)	0.02 ± 0.10 0.836*	−0.03 ± 0.08 0.247*	*−0.08 ± 0.07 * 0.048*	−0.05 ± 0.11 0.265*	−0.10 ± 0.11 0.072*	−0.05 ± 0.09 0.437*
100% (LE)	0.03 ± 0.14 0.730*	0.03 ± 0.10 0.694*	−0.12 ± 0.10 0.487*	0.00 ± 0.16 0.729*	−0.15 ± 0.15 0.757*	−0.15 ± 0.12 0.438*
10% (LE)	0.01 ± 0.09 **0.005** ^+^	0.04 ± 0.07 0.649*	0.00 ± 0.06 0.933*	0.03 ± 0.10 0.796*	−0.01 ± 0.09 0.814*	−0.04 ± 0.08 0.697*
100% (BE)	0.09 ± 0.08 0.389*	−0.08 ± 0.06 1.000^+^	0.05 ± 0.06 0.528*	−0.17 ± 0.09 0.005*	−0.04 ± 0.09 0.756*	0.12 ± 0.07 0.028*
10% BE	−0.07 ± 0.09 0.679*	−0.01 ± 0.07 0.945*	−0.04 ± 0.06 0.271*	0.00 ± 0.10 0.797*	−0.03 ± 0.10 0.696	−0.03 ± 0.08 0.381*

^+^One-way ANOVA (postHoc: bonferroni). *Paired independent samples (Mann-Whitney test).
